# Diverse electrical responses in a network of fractional-order conductance-based excitable Morris-Lecar systems

**DOI:** 10.1038/s41598-023-34807-3

**Published:** 2023-05-22

**Authors:** Sanjeev K. Sharma, Argha Mondal, Eva Kaslik, Chittaranjan Hens, Chris G. Antonopoulos

**Affiliations:** 1 Department of Mathematics, VIT-AP University, Amaravati, 522237 Andhra Pradesh India; 2 Department of Mathematics, Sidho-Kanho-Birsha University, Purulia, 723104 West Bengal India; 3 Department of Mathematical Sciences, University of Essex, Wivenhoe Park, Colchester, UK; 4 Department of Mathematics and Computer Science, West University of Timisoara, Timisoara, Romania; 5 Institute for Advanced Environmental Research, West University of Timisoara, Timisoara, Romania; 6 International Institute of Information Technology, Hyderabad, India

**Keywords:** Applied mathematics, Dynamical systems

## Abstract

The diverse excitabilities of cells often produce various spiking-bursting oscillations that are found in the neural system. We establish the ability of a fractional-order excitable neuron model with Caputo’s fractional derivative to analyze the effects of its dynamics on the spike train features observed in our results. The significance of this generalization relies on a theoretical framework of the model in which memory and hereditary properties are considered. Employing the fractional exponent, we first provide information about the variations in electrical activities. We deal with the 2D class I and class II excitable Morris-Lecar (M-L) neuron models that show the alternation of spiking and bursting features including MMOs & MMBOs of an uncoupled fractional-order neuron. We then extend the study with the 3D slow-fast M-L model in the fractional domain. The considered approach establishes a way to describe various characteristics similarities between fractional-order and classical integer-order dynamics. Using the stability and bifurcation analysis, we discuss different parameter spaces where the quiescent state emerges in uncoupled neurons. We show the characteristics consistent with the analytical results. Next, the Erdös-Rényi network of desynchronized mixed neurons (oscillatory and excitable) is constructed that is coupled through membrane voltage. It can generate complex firing activities where quiescent neurons start to fire. Furthermore, we have shown that increasing coupling can create cluster synchronization, and eventually it can enable the network to fire in unison. Based on cluster synchronization, we develop a reduced-order model which can capture the activities of the entire network. Our results reveal that the effect of fractional-order depends on the synaptic connectivity and the memory trace of the system. Additionally, the dynamics captures spike frequency adaptation and spike latency that occur over multiple timescales as the effects of fractional derivative, which has been observed in neural computation.

## Introduction

Neurons generate their diverse spike responses in different ways to the inputs. This shows important computational characteristics depending on the stimulus variance. Various electrical responses can be reproduced mathematically when we model the membrane voltage dynamics using coupled nonlinear ODEs with different suitable parameters and time scales^1–3^. Some excitable models exhibit spontaneous firing responses with multiple timescale dynamics, in particular the bursting behavior, consisting of periods of repetitive firing interspersed by quiescent phases^2^. The underlying mechanism of information processing depends on the cellular membrane voltages. However, a detailed description of diverse firing features and its characteristics cannot be revealed from a single neuron or coupled neurons using mathematical modeling. This shows a fundamental challenge in dynamical systems as the transition phases across different firing responses or the emergence of scale invariance in membrane voltage is always restricted^2^ due to various parameter regimes in neural computation. Recent research has been focused on fractional-order dynamics (FOD)^4–9^ in computational neurosciences, that can generate (depending on fractional order exponents) a wide range of firing phenomena or multiple timescale dynamics^10–21^. As such, a deeper understanding has been reached in different areas of biophysical processes^11,22–28^, showing more realistic dynamical features^29–33^. Fractional-order derivatives provide a mathematical framework in which memory dependent properties are considered. Earlier, a geometrical^7,34^ interpretation for the fractional-order derivative was introduced, which suggests that inhomogeneity of the time scale exists in the system. It may have an impact on the delays of signals or history dependent activities in comparison to the temporal order dynamics. In the fractional-order domain, the present state of the system is influenced by the previous states. History dependent spiking features are important, as the neuronal activities develop over time and continuously integrate the previous information^9,35^. Fractional-order neuronal models have been applied to study different firing responses^13,18,19,36,37^, firing rates and spike frequency adaptation both theoretically and experimentally. For some fractional-orders less than one, initially, the voltage increases faster, however, it reaches the steady state condition, i.e., quiescent state after longer time duration. Neurons can show adaptation in the fractional domain when we scale the input stimulus. The single spikes and various bursting maintain different information. The adaptation depends on the fractional exponent and the mean firing rates can change and adapt to the variations in the stimulus. The spike frequency adaptation follows power law dynamics in the fractional-order domain^10,18,38^.

The primary goal of the paper is to provide a brief description in understanding the effects of fractional-order derivative on the electrical activities of single M-L spiking neurons with class I & class II excitabilities and the slow-fast M-L neurons^1,2,39^ with its network architecture. One approach to study such firing characteristics and adaptation is to consider a conductance-based model that explores the intrinsic dynamics underlying the fractional-order derivatives. Previous works have investigated the various spiking responses depending on various parameter regimes, however, FOD can itself explore diverse firing responses^13,16^. The M-L models are taken into account for their diverse responses ranging from spiking to bursting. We consider different regimes in the parameter space of the M-L model: tonic spiking and fast spiking. Further, the model is extended to its 3D counterpart, where the applied current, *I* is not constant, but rather varies with time. We consider the fractional-order as the predominant parameter in the system and when it changes slowly, the spike transitions occur and we observe mixed-mode oscillations (MMOs) and mixed-mode bursting oscillations (MMBOs). It is one of the most interesting neuronal oscillations that emerge from the electrical activities^40,41^. MMOs are used to describe the alternating trajectories between small and large amplitude oscillations (SAOs and LAOs)^42,43^. These make the system fascinating and the output provides interesting and potential applications in a dynamical system. The emergence of MMBOs creates a spike adding mechanism. Earlier, it was observed that the MMOs reviewed the dynamical and neuronal behavior of locomotion or breathing^44,45^. It was observed in calcium signaling and electrocardiac systems^46,47^. Krupa et al.^48^ examined the mechanism of MMOs oscillations in a two-compartmental model of dopaminergic neurons in the mammalian brain stem. We also investigate the impact of electrical coupling on a mixed population where the neurons are either quiescent or oscillatory. Here, the neurons are assumed to be connected through the links of the Erdös-Rényi network. The coupling induces complex firing activities such as periodic bursting or spike frequency adaptation for all the nodes in the network. Based on the observed synchronization phenomena, a reduced-order model is developed which can produce the activities of the entire network.

In our work, we find consistent differences in the characteristics of the neuronal functional behavior using the fractional exponent. The fractional-order voltage dynamics can significantly change the spiking features of different single neuron models^12–15,17,18,36,37^. Realistic features can build the model more sensitive to neuronal dynamics, particularly in the potential collective behavior of the network, where past dynamical behavior might influence the present states.

## Formulation of the excitable model dynamics and some preliminaries

In this section, we describe the fractional-order excitable conductance-based model and review the existence of various characteristics observed in cortical areas^2^. We establish a particular parameter regime that supports the firing features with the variations of fractional exponent. To generate diverse spikes using fractional-order dynamics, we study the 2D and 3D M-L models with particular parameters and channel dynamics. Here, we choose the two models to separate the effects of fractional derivatives on the dynamical behavior of the model. Morris and Lecar^1,2^ proposed a simple mathematical model to describe the oscillations in the barnacle giant muscle fiber consisting of the membrane voltage equation with instantaneous activation of calcium current and an additional recovery equation describing slow activation of potassium current. The 2D M-L model is described in a commensurate fractional-order domain as follows

1 $$\begin{aligned} & C\frac{{{d^\alpha }u}}{{d{t^\alpha }}} = - 0.5g_{Ca}(u-V_{Ca})((1+\tanh (u-V_1))/V_2)-v{g_K}(u-V_K)-g_L(u-V_L)+I=h_1(u, v),\\ & \frac{{{d^\alpha }v}}{{d{t^\alpha }}}= \phi \cosh ((u-V_3)/2V_4)(0.5(1+\tanh ((u-V_3)/V_4))-v) =h_2(u, v). \end{aligned}$$

The biophysically motivated excitable model involves a voltage-gated $Ca^{2+}$ current, delayed rectifier $K^+$ current and a leak current respectively. *u* measures the membrane voltage dynamics and *v* is the activation variable of $K^+$ ion channels. The parameters ${g_{Ca}},\,\,{g_K}$ and ${g_L}$ indicate the maximum conductance functions to $C{a^{2 + }},\,\,{K^ + }$ and leak currents respectively. ${V_{Ca}}$, ${V_K}$ and ${V_L}$ are the reversal potentials to different ionic current functions. *C* measures the membrane capacitance. $\phi$ represents the temperature scaling factor for $K^+$ channel opening. The parameters ${V_1}$, ${V_2}$, ${V_3}$ and ${V_4}$ have fixed positive values. *I* indicates the applied stimulus. We would like to account the effects of various injected current stimulus on the fractional-order system with the fractional exponent, $\alpha$ ($0 < \alpha \le 1$).

### Slow-fast dynamical phenomenon

First, we assume the neuron is at the onset of firing and it generates spike generation as the control parameter moves slowly. The slow-fast dynamics can be mathematically modeled as^1^

2 $$\begin{aligned} \begin{array}{l} \dot{x}(t) = f(x,\,\,z),\\ \dot{z}(t) = \delta g(x,\,\,z), \end{array} \end{aligned}$$

where $\dot{x}(t) = f(x,\,z)$ (fast spiking) and $\dot{z}(t) = \delta g(x,\,z)$ (slow modulation). $x \in \mathbb {R} {^m}$ represents the fast variables and $z \in \mathbb {R}{^n}$ the slow variables with $0< \delta< < 1$ measuring the timescale separation parameter.

The following system of ODEs represents the slow-fast 3D M-L model where (*u*, *v*) denote the fast subsystem and *w* slow variable. The fractional-order modified 3D M-L model^1,13^ is presented as

3 $$\begin{aligned} & C\frac{{{d^\alpha }u}}{{d{t^\alpha }}} = - 0.5g_{Ca}(u-1)((1+\tanh (u-V_1))/V_2)-v{g_K}(u-V_K)-g_L(u-V_L)+I(w)=f_1(u, v, w),\\ & \frac{{{d^\alpha }v}}{{d{t^\alpha }}}= \phi \cosh ((u-V_3)/2V_4)(0.5(1+\tanh ((u-V_3)/V_4))-v) =f_2(u, v, w),\\ & \frac{{{d^\alpha }w}}{{d{t^\alpha }}}= \mu (V_0+u)=f_3(u, v, w). \end{aligned}$$

The system has the following characteristics. The system variable *w* is the external injected current which follows the power law dynamics in the fractional system and characterizes the memory effect of the membrane potential^1,13,16^. The parameters ${V_1},\,\,{V_2},\,\,{V_3}\,\,\mathrm{{and}}\,\,{V_4}$ are suitably selected for the hyperbolic functions in order to explain that they can reach their equilibrium points instantaneously. The parameter value $\mu$ is less than 1 i.e., $0< \mu < 1$ which measures the ratio of time scale between oscillations and modulation. Lundstrom et al.^10^ studied that pyramidal neurons can act as fractional differentiators of the stimulus amplitude envelope for this type of input. FOD can generalize the derivative operator such that, to obtain the first order derivative of a function, differentiate twice taking the fractional-order derivative of order $\alpha = 1/2$ and it results in the first derivative^4,6^. It filters the response with a decaying time constant that depends on $\alpha$.

The parameter sets for all the simulation results are considered as (for Eq. 1)^1,39^ Set I: $C = 20,\,\,{g_{Ca}} = 4,\, g_K=8,\,g_L=2,\,V_{Ca}=120,\,V_K=-84,\,V_L=-60,\,{V_1} = -1.2,\,{V_2} = 18,\,{V_3} = 12,\,{V_4} = 17.4,\,\,\phi = 0.067$ (for class I excitable membrane model) with varying *I*, Set I: $I=40$, Set II: $I=45$ and for the class II membrane model, the parameters are the same described above except Set III: ${g_{Ca}} = 4.4,\, {V_3} = 2,\,{V_4} = 30,\,\,\phi = 0.04$, and $I=100$. In order to study the system dynamics, we first analyze the equilibrium states and then bifurcations. Next, we use the following sets of parameters to deal with system (3) and its modified versions by considering $I(w)=0.08-0.03w$ using $C=1$ for the parameter sets I, II and III respectively.

Set I: $$g_\mathrm{Ca}=0.9,\,g_\mathrm{K}=2,\,g_L=0.5,\,V_{Ca}=1,\, V_K=-0.7,\, V_L=-0.5,\, V_1=-0.01,\,V_2=0.15,\, V_3(w)=(0.08-w),\,V_4=0.04,\, \phi =1/3,\,\mu =0.003,\, V_0=0.22$$

Set II: $$g_\mathrm{Ca}=1.36,\,g_\mathrm{K}=2,\,g_L=0.5,\,V_{Ca}=1,\, V_K=-0.7,\, V_L=-0.5,\, V_1=-0.01,\,V_2=0.15,\, V_3(w)=(0.08-w),\,V_4=0.16,\, \phi =1/3,\,\mu =0.003,\, V_0=0.1$$

Set III: $$g_\mathrm{Ca}=0.9,\,g_\mathrm{K}=2,\,g_L=0.5,\,V_{Ca}=1,\, V_K=-0.7,\, V_L=-0.5,\, V_1=-0.01,\,V_2=0.15,\, V_3(w)=(0.08-w),\,V_4=0.05,\, \phi =1/3,\,\mu =0.005,\, V_0=0.1$$

## Preliminaries to systems of fractional-order differential equations

To study the fractional-order M-L model, we consider the familiar definition of the fractional derivative i.e., the Caputo fractional-order derivative^6,34^. The commensurate fractional-order model with fractional exponent $\alpha \in (0, 1)$ can be described as

4 $$\begin{aligned} D^\alpha \text {X} = f\left( {X} \right) , \end{aligned}$$

where either $X(t)=(u(t), v(t))\in \mathbb {R}^2$ or $X(t)=(u(t), v(t), w(t))\in \mathbb {R}^3$, and $f=(h_1, h_2)$ or $f=(f_1, f_2, f_3)$ for 2D and 3D cases, respectively. The Caputo fractional differential operator is defined as

5 $$\begin{aligned} D^\alpha X (t) =\frac{d^\alpha X}{dt^\alpha }= \frac{1}{{\Gamma \left( {1 - \alpha } \right) }}\int \limits _0^t {\mathop {\left( {t - \tau } \right) }\nolimits ^{ - \alpha } {X}'\left( \tau \right) d\tau }, \end{aligned}$$

where the Gamma function is given by $\Gamma \left( z \right) = \int \nolimits _0^\infty {{e^{ - s}}{s^{z - 1}}ds}$. The limits of the integration (i.e., from 0 to *t*) show that, in contrast with the classical integer-order derivative, the fractional-order derivative depends on the whole previous history of the function. Hence, due to the non-locality of the Caputo differential operator, a fractional-order mathematical model is able to reflect memory properties of the system variables. It is important to note that for $\alpha =1$, the Caputo derivative converges to the first-order integer derivative. An additional advantage of Caputo-type fractional -order derivative over other types of fractional differential operators is that the derivative of a constant is zero. It is efficient to integrate all the previous activities of the function weighted by a function that follows power-law dynamics^9,14,15^.

### Remark 3.1

 In the investigation of the local stability properties of an equilibrium of a dynamical system, the classical Hartman-Grobman linearization theorem plays a fundamental role: it states that the local behavior of a dynamical system in a neighborhood of a hyperbolic equilibrium is qualitatively equivalent to the behavior of its linearization near the equilibrium. It is important to remark that a fractional-order counterpart of this linearization theorem has been obtained in^30^. If $X^\star$ is an equilibrium of system system (4), i.e. $f(X^\star )=0$, the corresponding linearized system at $X^\star$ is:

6 $$\begin{aligned} D^\alpha X = J_f(X^\star )X~, \end{aligned}$$

where $J_f(X^\star )$ is the Jacobian matrix of the function *f* computed at $X^\star$. Therefore, the equilibrium $X^\star$ of the nonlinear system (4) is asymptotically stable if and only if the trivial solution of the linearized system (6) is asymptotically stable^49–51^. Furthermore, based on the well-known Matignon’s theorem^49^, the linearized fractional-order system (6) is asymptotically stable if and only if $|\arg (\lambda )|>\frac{\alpha \pi }{2}$, for any eigenvalue $\lambda$ of the Jacobian matrix $J_f(X^\star )$.

### Definition 3.1

If some eigenvalues of the Jacobian matrix $J_f(X^\star )$ satisfy $|\arg (\lambda )|>\frac{\alpha \pi }{2}$ and some other eigenvalues satisfy $|\arg (\lambda )|<\frac{\alpha \pi }{2}$, then the equilibrium $X^\star$ is a called a *saddle point*^52,53^.

### Remark 3.2

In a 3D nonlinear fractional-order system, an equilibrium $X^\star$ is called a *saddle of index one* if one of the eigenvalues of the Jacobian matrix $J_f(X^\star )$ is unstable (i.e. $|\arg (\lambda _1)|<\frac{\alpha \pi }{2}$) and other two eigenvalues are stable $|\arg (\lambda _{2,3})|>\frac{\alpha \pi }{2}$. On the other hand, if two eigenvalues associated to the equilibrium $X^\star$ are unstable, while only one eigenvalue is stable, the saddle point $X^\star$ is called *saddle of index two*^53^.

We numerically simulated the model (Eqs. 1 and 3) using the L1 scheme^4,12,18^ and approximated the fractional-order derivative as described in Appendix.

## Qualitative analysis and theoretical considerations

### Analysis of the 2D system

System (1) is a particular case of the 2D fractional-order conductance-based excitable model:

7 $$\begin{aligned} \left\{ \begin{array}{rl} C\cdot D^\alpha u(t)&{}=I-\tilde{I}(u,v),\\ D^\alpha v(t)&{}=\phi \ell (u)(v_\infty (u)-v), \end{array}\right. \end{aligned}$$

where *u* and *v* are the membrane voltage and the gating variable of the neuron, *I* is an applied current, $\tilde{I}(u,v)$ represents the ionic current, $\ell (v)$ is the rate constant for opening ionic channels and $v_\infty (v)$ represents the fraction of open ionic channels at steady state, respectively.

In particular, we have from model (1):

8 $$\begin{aligned} \tilde{I}(u,v)=g_{Ca}m_\infty (u)(u-1)+g_K\cdot v(u-V_K)+g_L(u-V_L), \end{aligned}$$

and

$$\begin{aligned} m_\infty (u)=\frac{1}{2}\Big (1+\tanh \left( \frac{u-V_1}{V_2}\right) \Big )\quad ,\quad v_{\infty }(u)=\frac{1}{2}\left( 1+\tanh \left( \frac{u-V_3}{V_4} \right) \right) ,\quad \ell (u)=\cosh \Big (\frac{u-V_3}{2V_4}\Big ).\end{aligned}$$

The equilibrium points of system (7) are the solutions of the algebraic system:

$$\begin{aligned}I=\tilde{I}(u,v),\quad v=v_\infty (u),\end{aligned}$$

which is equivalent to

$$\begin{aligned} I=\tilde{I}(u,v_\infty (u)):=I_\infty (u),\quad v=v_\infty (u). \end{aligned}$$

The function $I_\infty (u)$ satisfies the following basic properties:

- $$I_\infty \in C^1(\mathbb {R})$$;
- $$\lim \limits _{u\rightarrow -\infty }I_\infty (u)=-\infty$$ and $$\lim \limits _{u\rightarrow \infty }I_\infty (u)=\infty$$;
- $I'_{\infty }$ has exactly two real roots $u_{max}<u_{min}$.

Denoting by $I_{max}=I_\infty (u_{max})$ and $I_{min}=I_\infty (u_{min})$ the maximum and minimum values of $I_\infty$, respectively, the function $I_\infty$ is increasing on the intervals $(-\infty , u_{max}]$ and $[u_{min}, \infty )$ and decreasing on the interval $(u_{max}, u_{min})$.

Hence, depending on the external input *I*, there are exactly three branches of equilibrium points, denoted by $(u_i(I),v_\infty (u_i(I)))$, $i\in \{1,2,3\}$, where:

$$\begin{aligned}&I_1=I_\infty |_{(-\infty , u_{max}]}, \quad u_1:(-\infty , I_{max}]\rightarrow (-\infty , u_{max}],\quad u_1(I)=I_1^{-1}(I)\\&I_2=I_\infty |_{(u_{max},u_{min})},\quad u_2:(I_{min},I_{max})\rightarrow (u_{max}, u_{min}),\quad u_2(I)=I_2^{-1}(I)\\&I_3=I_\infty |_{[u_{min}, \infty )},\quad u_3:[I_{min}, \infty )\rightarrow [u_{min}, \infty ),\quad u_3(I)=I_3^{-1}(I) \end{aligned}$$

Consequently, one of the following situations may hold:

- If $I<I_{min}$ or if $I>I_{max}$, then system (1) has a unique equilibrium point.
- If $I=I_{min}$ or if $I=I_{max}$, then system (1) has two equilibrium points.
- If $I\in (I_{min},I_{max})$, then system (1) has three equilibrium points.

The Jacobian matrix associated to the system (1) at an arbitrary equilibrium state $(u^\star ,v^\star )=(u^\star , v_\infty (u^\star ))$ is:

$$\begin{aligned} J=\begin{bmatrix} -\tilde{I}_u(u^\star ,v_{\infty }(u^\star ))/C &{} -\tilde{I}_v(u^\star ,v_{\infty }(u^\star ))/C\\ \phi \ell (u^\star )v'_\infty (u^\star )&{} -\phi \ell (u^\star ) \end{bmatrix}. \end{aligned}$$

In this case, the necessary and sufficient conditions for the asymptotic stability of an equilibrium point $(u^\star ,v^\star )$ reduce to the following inequalities^54^:

$$\begin{aligned} \delta (u^\star )>0\quad \text {and}\quad \tau (u^\star )<2\sqrt{\delta (u^\star )}\cos \left( \frac{\alpha \pi }{2}\right) , \end{aligned}$$

where

$$\begin{aligned} \tau (u^\star )&=\text {trace}(J)=-\frac{1}{C}\tilde{I}_u(u^\star ,v_\infty (u^\star ))-\phi \ell (u^\star ),\\ \delta (u^\star )&=\det (J)= \frac{\phi }{C}\ell (u^\star )[\tilde{I}_u(u^\star , v_\infty (u^\star ))+v'_\infty (u^\star )\cdot \tilde{I}_v(u^\star , v_\infty (u^\star ))] =\frac{\phi }{C}\ell (u^\star )I'_\infty (u^\star ). \end{aligned}$$

We first remark that the second branch of equilibrium points is completely unstable. Indeed, any equilibrium point $(u_2(I),v_\infty (u_2(I)))$ with $I\in (I_{min},I_{max})$, satisfies $I'_\infty (u_2(I))<0$, and hence, $\delta (u_2(I))<0$. In fact, the negative sign of the Jacobian’s determinant guarantees that each equilibrium point of the second branch is a saddle point, no matter what fractional-order $\alpha$ is considered in system (7).

On the other hand, along the first and third branches of equilibrium points, it is easy to find that the determinant of Jacobian is positive. Hence, the stability of the equilibrium points particularly depends on the trace $\tau$. Obviously, if $\tau (u^\star )<0$, the equilibrium point $(u^\star ,v^\star )$ is asymptotically stable, irrespective of the fractional-order $\alpha$ considered in system (7). However, if $\tau (u^\star )\ge 0$, an equilibrium point $(u^\star ,v^\star )$ of the first or the third branch is asymptotically stable, if and only if

9 $$\begin{aligned} \alpha <\alpha ^\star (u^\star )=\frac{2}{\pi }\arccos \left( \frac{\tau (u^\star )}{2\sqrt{\delta (u^\star )}}\right) . \end{aligned}$$

We will further assume that $V_k<u_{max}<u_{min}<1$. We can easily evaluate:

$$\begin{aligned} \tilde{I}_u(u,v_\infty (u))=g_{Ca}[m'_{\infty }(u)(u-1)+m_{\infty }(u)]+g_k\cdot v_\infty (u)+g_L, \end{aligned}$$

and hence, if $(u^\star ,v^\star )=(u^\star ,v_\infty (u^\star ))$ is an equilibrium point of the third branch such that $u^\star >1$, it follows that $\tilde{I}_u(u^\star ,v_\infty (u^\star ))>0$, and hence $\tau (u^\star )<0$.

Furthermore, we can also express

$$\begin{aligned} \tilde{I}_u(u,v_\infty (u))=I_\infty '(u)-v_\infty '(u)\tilde{I}_v(u,v_\infty (u))=I_\infty '(u)-v_\infty '(u)\cdot g_k(u-V_K), \end{aligned}$$

and hence, if $(u^\star ,v^\star )$ is an equilibrium point of the first branch such that $u^\star <V_K$, we deduce that $\tilde{I}_u(u^\star ,v_\infty (u^\star ))>0$, and similarly as above, we get $\tau (u^\star )<0$.

Based on the above calculation, we also remark that:

$$\begin{aligned} \tau (u_{m})=-\frac{1}{C}\left[ I_\infty '(u_{m})-v_\infty '(u_{m})\cdot g_k(u_{m}-V_K)\right] -\phi \ell (u_{m})=\frac{1}{C}v_\infty '(u_{m})\cdot g_k(u_{m}-V_K)-\phi \ell (u_{m}), \end{aligned}$$

for either $u_m=u_{max}$ or $u_m=u_{min}$, and assuming that $\phi$ is small enough, it can be observed that the inequality $\tau (u_m)>0$ might hold. Therefore, the function $\tau (u)$ might have two roots $u'\in (V_K,u_{max})$ and $u''\in (u_{min},1)$, respectively. Based on the numerical data, we can further assume that if they exist, these roots are unique in the aforementioned intervals.

In conclusion, the stability of equilibrium states may depend on the fractional-order $\alpha$ only in the following two cases:

- the equilibrium point belongs to the first branch and $u^\star \in (u',u_{max})$;
- the equilibrium point belongs to the third branch and $u^\star \in (u_{min},u'')$.

In this case, the critical value $\alpha ^\star$ given by (9) corresponds to a Hopf-type bifurcation (i.e. the Jacobian matrix has a pair of complex conjugate eigenvalues such that $|\arg (\lambda )|=\frac{\alpha \pi }{2}$). In other words, the position of the Hopf bifurcation points in the (*I*, *u*)-plane, situated on the first and / or third branches, respectively, depending on the fractional-order $\alpha$ considered in system (7). Obviously, this will have a direct effect on the type of spiking and bursting behavior both in the 2D system (7) as well as in the 3D slow-fast system, as it will be unveiled in the next section.

**Figure 1:**
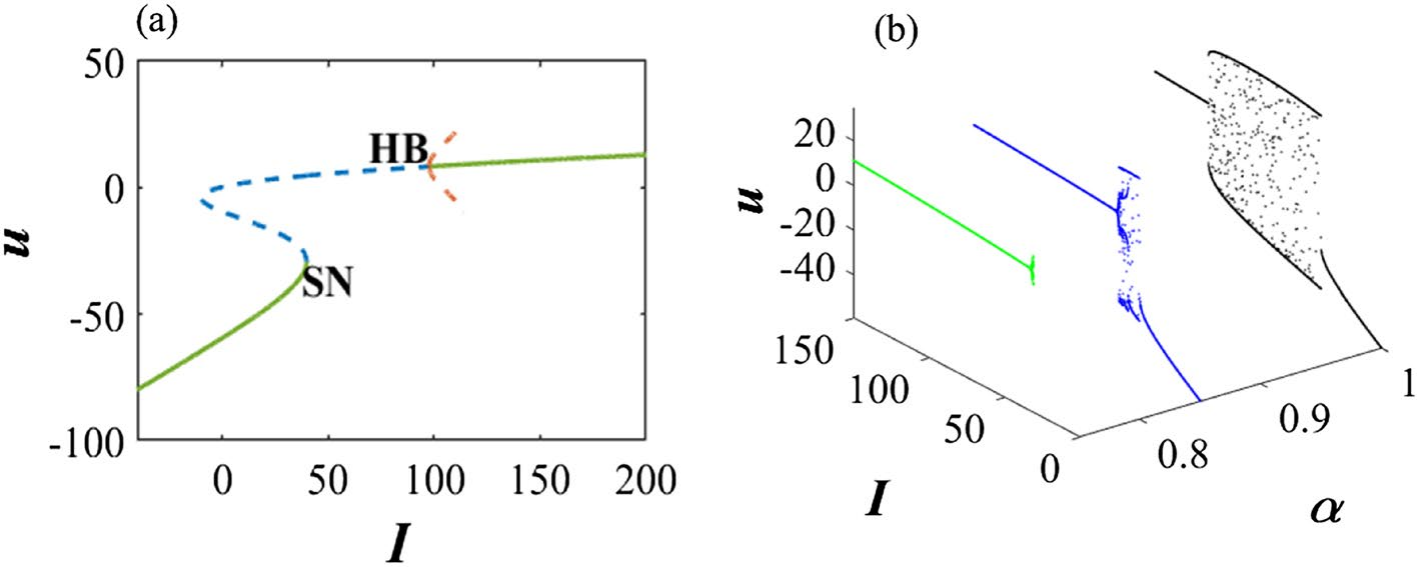
Bifurcation scenarios of the 2D M-L model (1) for set I and set II. (**a**) *I* as a bifurcation parameter: HB ($I=97.65$ for $\alpha =1$) and SN ($I=39.96$) represent the existence of Hopf bifurcation and saddle-node bifurcation in the system (1). The solid green lines and dotted blue line indicate the stable and unstable equilibrium branch of the system respectively. However, the dotted brown line represents the emergence unstable limit cycle at HB. (**b**) *I* and $\alpha$ as bifurcation parameters: green, blue and black curves represent the dynamics of the system (1) at fractional orders 0.75, 0.85, and 1, respectively.

**Figure 2:**
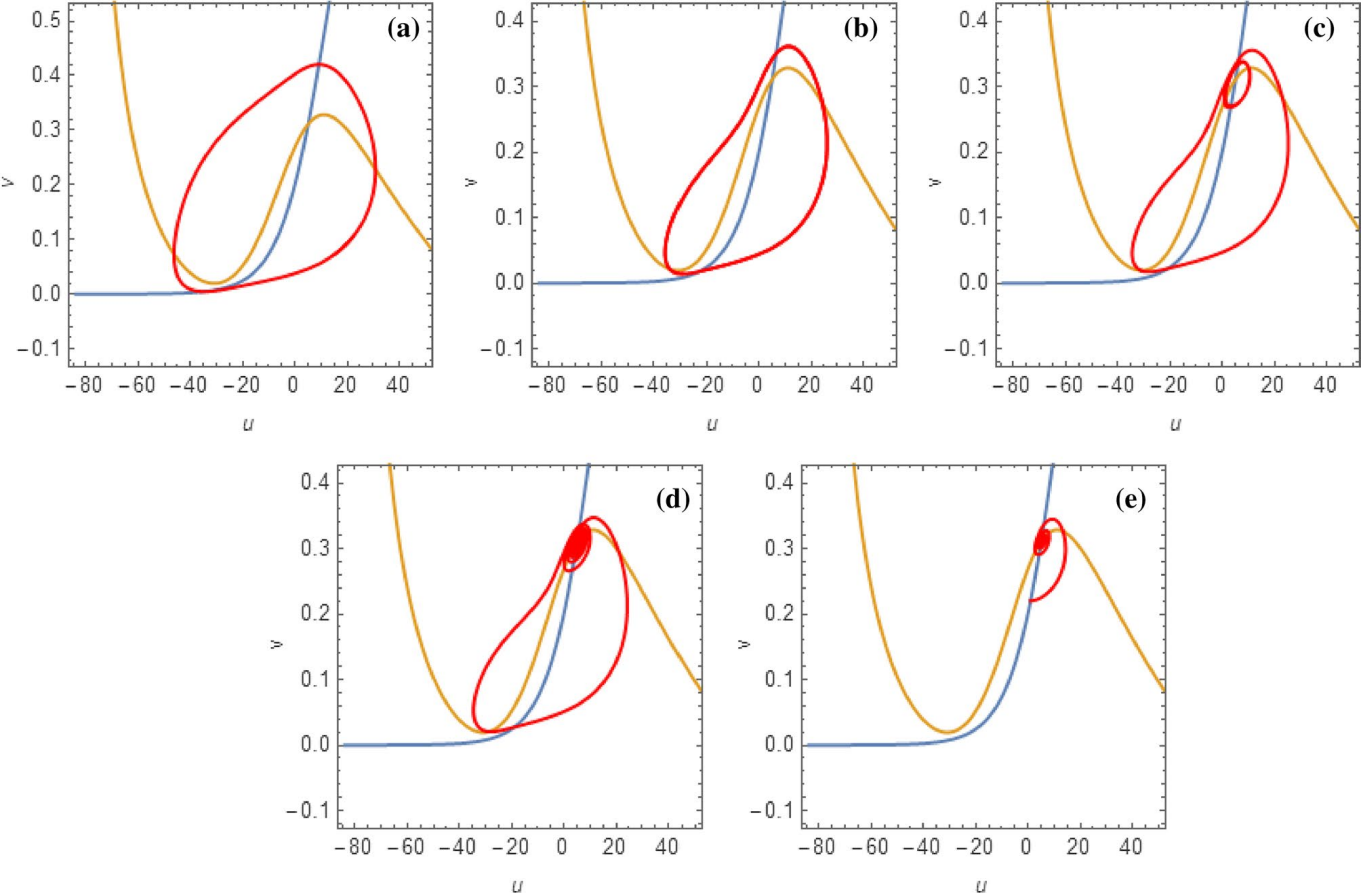
Phase portraits (including nullclines) in the (*u*, *v*)-plane for the 2D Morris-Lecar model for $g_{Ca} = 4$, $V_{Ca} = 120$, $V_1 = -1.2$, $V_2 =18$, $g_K = 8$, $V_K = -84$, $g_L = 2$, $V_L = -60$, $\phi = 0.067$, $V_3 = 12$, $V_4 = 17.4$, $C = 20$, $I = 45$ with the fractional-orders (from left to right): (**a**) $\alpha =1$; (**b**) $\alpha =0.85$; (**c**) $\alpha =0.83$; (**d**) $\alpha =0.8$; (**e**) $\alpha =0.78$, respectively.

**Figure 3:**
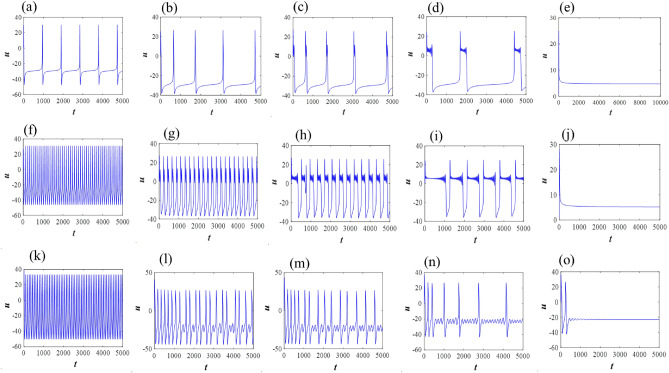
Time series of class I & class II excitable 2D single M-L model (1) for different fractional exponents, (**a**–**e**) $\alpha =1$, 0.85, 0.83, 0.80, 0.75 with $I=40$; (**f**–**j**) $\alpha =1$, 0.84, 0.82, 0.80, 0.75 with $I=45$ (parameter sets I and II); and (**k**–**o**) $\alpha =1$, 0.86, 0.85, 0.84, 0.81 with $I=100$ (parameter set III).

As an example, first we show the bifurcation scenario of the classical 2D M-L model with Hopf bifurcation points by considering *I* as a bifurcation parameter (Fig. 1a). Next, we consider the effect of fractional order on the dynamics of system (1) and showed that how it stabilizes the system as we decrease the value of $\alpha$ (Fig. 1b). Then, Fig. 2 presents the phase portraits of the 2D M-L model with the parameters from Set II, for different values of the fractional-order $\alpha$. In this case, there is only one unstable equilibrium point for the system, namely $(u^\star ,v^\star )=(5.08955, 0.311245)$ (at the intersection of the nullclines), situated on the third branch. The critical value of the fractional-order corresponding to the Hopf bifurcation at the equilibrium point is $\alpha ^\star =0.787825$, computed by the formula (9). In the integer-order case, $\alpha =1$, a large-amplitude limit cycle attractor is present, corresponding to spiking behavior. As the fractional-order $\alpha$ decreases, the large-amplitude attractive quasi-periodic limit cycle approaches the unstable equilibrium point and as $\alpha$ approaches the critical value $\alpha ^\star$ for Hopf bifurcation, a more complex quasi-periodic orbit emerges, involving smaller-amplitude oscillations around the equilibrium, as well as large-amplitude spikes. When $\alpha <\alpha ^\star$, the equilibrium becomes asymptotically stable. In Fig. 3, we show corresponding time series to further verify the numerical results, noting that the computed critical values of the fractional-order are $\alpha ^\star =0.757245$ for Set I and $\alpha ^\star =0.834537$ for Set III, respectively.

### Analysis of the 3D system

In line with the previously presented aspects, the 3D slow-fast fractional-order model (3) can be written as:

10 $$\begin{aligned} \left\{ \begin{array}{rl} C\cdot D^\alpha u(t)&{}=I(w)-\tilde{I}(u,v),\\ D^\alpha v(t)&{}=\phi \tilde{\ell }(u,w)(\tilde{v}(u,w)-v),\\ D^\alpha w(t)&{}=\mu (u+V_0), \end{array}\right. \end{aligned}$$

where $\tilde{I}(u,v)$ is given by (8) and

$$\begin{aligned} \tilde{v}(u,w)=\frac{1}{2}\left( 1+\tanh \left( \frac{u-V_3(w)}{V_4} \right) \right) ,\quad \tilde{\ell }(u,w)=\cosh \Big (\frac{u-V_3(w)}{2V_4}\Big ). \end{aligned}$$

We will assume that *I*(*w*) and $V_3(w)$ are decreasing functions, and that $V_0+V_K<0$ (according to the considered parameter sets). The unique equilibrium point of system (10) is $(u^\star ,v^\star ,w^\star )$, where $u^\star =-V_0$, $v^\star =\tilde{v}(u^\star ,w^\star )$, and $w^\star$ is the unique root of the strictly decreasing function $w\mapsto I(w)-\tilde{I}(-V_0,\tilde{v}(-V_0,w))$.

The Jacobian matrix at the equilibrium point $(u^\star ,v^\star ,w^\star )$ is

$$\begin{aligned} J=\begin{bmatrix} -\tilde{I}_u(u^\star ,v^\star )/C &{} -\tilde{I}_v(u^\star ,v^\star )/C &{} I'(w^\star )/C\\ \phi \tilde{\ell }(u^\star ,w^\star )\tilde{v}_u(u^\star ,w^\star )&{} -\phi \tilde{\ell }(u^\star ,w^\star ) &{} \phi \tilde{\ell }(u^\star ,w^\star )\tilde{v}_w(u^\star ,w^\star )\\ \mu &{} 0 &{} 0 \end{bmatrix}. \end{aligned}$$

and its charactersitic equation is:

$$\begin{aligned} \lambda ^3+a\lambda ^2+b\lambda +c=0, \end{aligned}$$

where

$$\begin{aligned} a&=\frac{1}{C}\tilde{I}_u(u^\star ,v^\star )+\phi \tilde{\ell }(u^\star ,w^\star ),\\ b&=\frac{\phi }{C}\tilde{\ell }(u^\star ,w^\star )\left[ \tilde{I}_u(u^\star ,v^\star )+\tilde{I}_v(u^\star ,v^\star )\tilde{v}_u(u^\star ,w^\star )\right] -\frac{\mu }{C}I'(w^\star ),\\ c&= \frac{\mu \phi }{C} \tilde{\ell }(u^\star ,w^\star )\left[ \tilde{I}_v(u^\star ,v^\star )\tilde{v}_w(u^\star ,w^\star )-I'(w^\star )\right] >0. \end{aligned}$$

The positivity of the coefficient *c* follows from

$$\begin{aligned} \tilde{I}_v(u^\star ,v^\star )=g_k(u^\star -V_K)=-g_k(V_0+V_k)>0. \end{aligned}$$

As $c>0$, it follows that the product of the eigenvalues of the Jacobian matrix is negative, and hence, one of the eigenvalues is a negative real number and the other two eigenvalues are either complex conjugated, or are real and have the same sign. We will further assume that at least one of the coefficients *a* or *b* is negative (based on the parameter sets under consideration), and hence, it is clear that the Routh-Hurwitz conditions are not satisfied for the characteristic polynomial. Hence, denoting by $\Delta$ the discriminant of the characteristic polynomial, we distinguish two cases:

- if $\Delta >0$, the Jacobian matrix *J* has one negative and two positive eigenvalues, and consequently, the equilibrium point $(u^\star ,v^\star ,w^\star )$ is a saddle point of index two, for any fractional-order $\alpha$ (e.g. in the case of parameters from Set I and Set II);
- if $\Delta <0$, the Jacobian matrix *J* has one negative eigenvalue and two complex conjugate eigenvalues with positive real part (e.g. in the case of parameters from Set III). Consequently, there exists a critical value $\alpha ^\star$ of the fractional-order such that the equilibrium point $(u^\star ,v^\star ,w^\star )$ is asymptotically stable for $\alpha <\alpha ^\star$ and unstable for $\alpha >\alpha ^\star$. At $\alpha =\alpha ^\star$, a Hopf-type bifurcation occurs in a neighborhood of the equilibrium point, resulting in the appearance of persistent oscillations. The critical value $\alpha ^\star$ is found using the method presented in^55^ ($\alpha ^\star =0.62477$ for Set III).

## Analysis of diverse oscillatory responses

We start our discussion with the fractional-order class I and class II single M-L neurons and then extend it to the slow-fast dynamics. We simulated the spikes from the single model, and the membrane voltage dynamics depends on the voltage-gated conductances. The input stimulus is considered as *I*. We tuned the fractional-order exponent, $\alpha$, with different parameter regimes: tonic spiking and fast spiking zone for class I and class II excitabilities. We next show the modulations of the electrical activities for a long time scale and the spike frequency adaptive effects. We considered two different suitable current stimuli, $I=40$ and 45 for class I neuron and $I=100$ for class II case. We choose these types of input stimuli as it shows the tonic spiking and fast spiking for the classical-order dynamics, however, when we change it in the fractional domain, the dynamical model produces variations in the firing features not explored earlier to the best of our knowledge. The bifurcation analysis is performed and the numerical results are supported by the stability analysis and there is a good agreement between the analytical and numerical findings.

First, we consider the class I excitable M-L neuron with parameter sets I and II. The classical-order neuron shows tonic spiking when stimulated, when the input stimulus current is on ($I=40$), the neuron continues to exhibit a train of spikes, called tonic spiking. Then, as the fractional exponent decreases to $\alpha =0.85$, it shows tonic spiking however, the interspike interval increases, i.e., firing frequency decreases. With further decrease of $\alpha =0.83$ and 0.80, it generates regular bursting and then regular bursting with low firing frequency. Then, it goes to quiescent state with a lower fractional exponent $\alpha =0.75$, which is in good agreement with analytical results (see Fig. 3a–e). Next, with parameter set II, the integer order single neuron shows fast spiking while the input stimulus is on $I=45$. With the decrease of $\alpha =0.84$, the firings transform into regular bursting, then with $\alpha =0.82$ and 0.80, it produces bursting however, the firing frequency decreases and more burst produces. Finally, it switches to quiescent state at $\alpha =0.75$ (see Fig. 3f–j).

Class II excitable neurons cannot generate low-frequency spikes. They are either in quiescent states or fire a train of spikes with larger frequency by a strong input current. The single M-L neuron with parameter set III shows fast spiking with $\alpha =1$ for $I=100$. With the decrease of $\alpha =0.86$ and 0.85, it generates MMBOs and MMOs. The firings switch to regular MMOs with further decrease of $\alpha =0.84$, however it shows MMOs with lower firing frequency, i.e., the inter spike interval increases. Then, it goes to quiescent state $\alpha =0.81$, i.e., converges to the fixed point of the system (see Fig. 3k–o).

**Figure 4:**
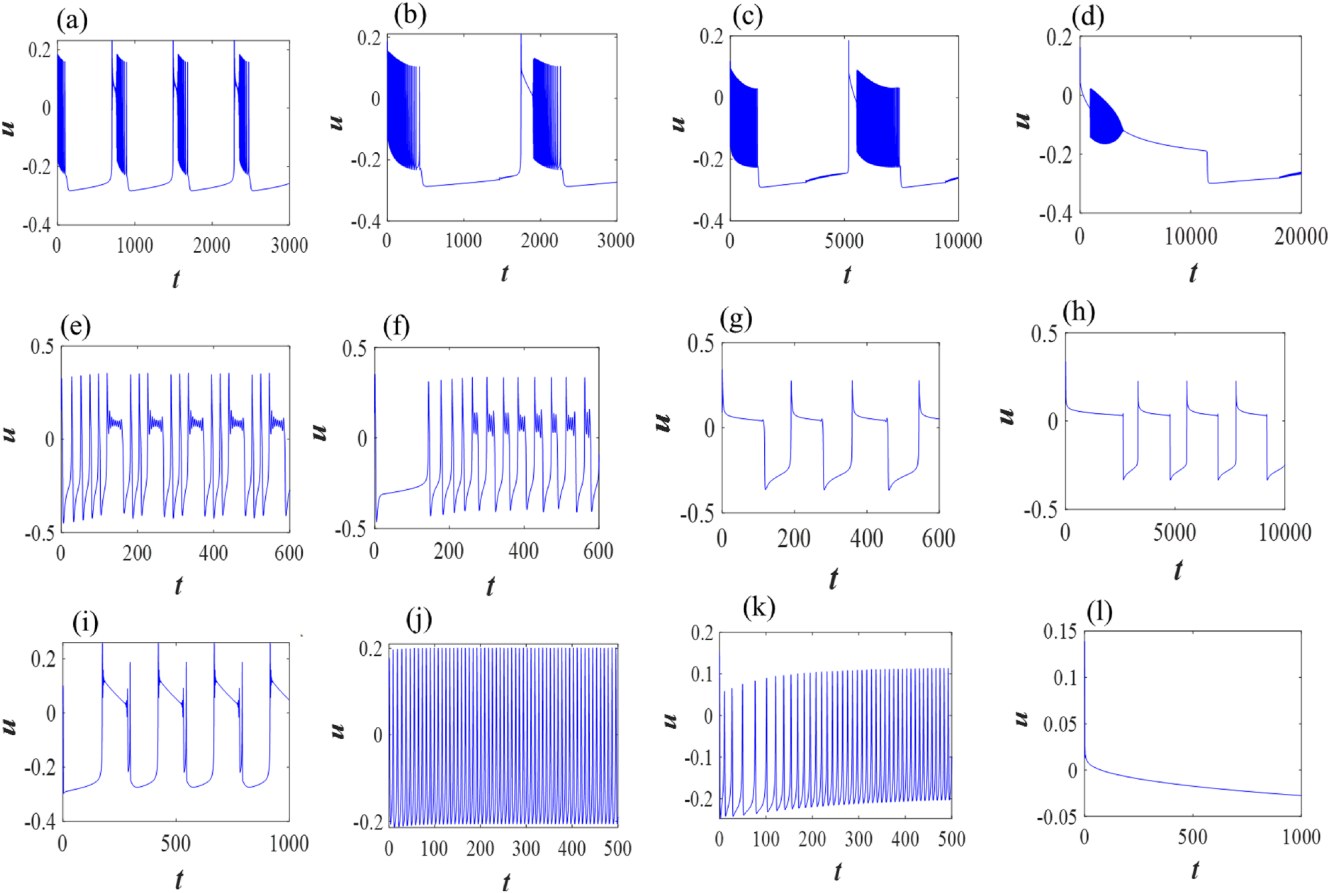
Time series of slow-fast excitable 3D single M-L model (3) for different fractional exponents, (**a**–**d**) $\alpha =1$, 0.9, 0.8, 0.7; (**e**–**h**) $\alpha =1$, 0.95, 0.75, 0.5; and (**i**–**l**) $\alpha =1$, 0.98, 0.8, 0.6 (parameter sets I, II and III).

Now, we extend our study with the excitable slow-fast single 3D M-L neuron model (3) in the fractional domain with various parameter regimes that generate different bursting features, i.e., the number of spikes in each burst varies with diverse small and large amplitudes. With parameter set I, the single M-L model at $\alpha =1$ produces bursting with several number of spikes in each burst, however with the decrease of $\alpha =0.9$ and 0.8, the firing frequency decreases with longer time period, i.e., interspike interval increases between two burst and the amplitude of each spike decreases in the simultaneous bursting. For this parameter set, the unique fixed point is a saddle point of index two. It is observed that spike frequency adaptation occurs with the decrease of fractional-order exponents. Then it generates more spike frequency adaptation with further decrease of $\alpha =0.7$ (see Fig. 4a–d). Similarly, with parameter set II, the classical single neuron model shows bursting. With decreasing $\alpha =0.95$ and 0.75, it shows various bursting and then spiking behavior is observed with spike frequency adaptation and first spike latency at $\alpha =0.5$ (see Fig. 4e–h). Finally, for set III, the single neuron changes it behavior from bursting to fast spiking while $\alpha$ changes from one to $\alpha =0.98$ and 0.8. It switches to stable steady state with $\alpha =0.6$, i.e., it converges to the locally asymptotically fixed point of the system (see Fig. 4i–l).

## Network analysis of the fractional-order 2D M-L model

We investigate various dynamics of fractional-order M-L model in a random network architecture, where all the neurons are connected randomly with each other and with connection probability $\tilde{p}$. We construct an Erdös-Rényi network^56,57^ of $N=100$ M-L oscillators that is considered with mean node-degree $\langle k \rangle \sim 7$ for numerical simulations. The elaborate discussion of the network architecture is explained in the following subsections.

### Dynamics of the network with two subpopulations

To capture different firing activities of the network, first we consider a heterogeneous network of two different subpopulations depending on fractional-order $(\alpha )$. Further, we consider that the fractional-order M-L neurons are electrically coupled through first state variable (*u*). The dynamics of network is studied using the following mathematical model

11 $$\begin{aligned} \begin{array}{l} C\frac{d^{\alpha _i}u_i}{dt^{\alpha _i}} =- 0.5g_{Ca}(u_i-V_{Ca})((1+\tanh (u_i-V_1))/V_2)-v_i{g_K}(u_i-V_K)-g_L(u_i-V_L)+I+\frac{g_e}{\sum _{j=1}^{N} c_{ij}}\sum _{j=1}^{N} c_{ij}(u_j - u_i),\\ \frac{d^{\alpha _i}v_i}{d t^{\alpha _i}} =\phi \cosh ((u_i-V_3)/2V_4)(0.5(1+\tanh ((u_i-V_3)/V_4))-v_i), \quad i= 1, 2, 3, \ldots , N. \end{array} \end{aligned}$$

The electrical coupling of the network is given by $g_e>0$. The connection matrix of the network is represented by $M=\left( c_{ij}\right) _{N\times N}$. Further, we divide the population of size *N* into two subpopulations depending on fractional-order as

$$\begin{aligned} \alpha _i=\left[ {\underbrace{\alpha ,\ldots \alpha ,}_m\underbrace{\beta ,\ldots \beta }_n} \right] . \end{aligned}$$

**Figure 5:**
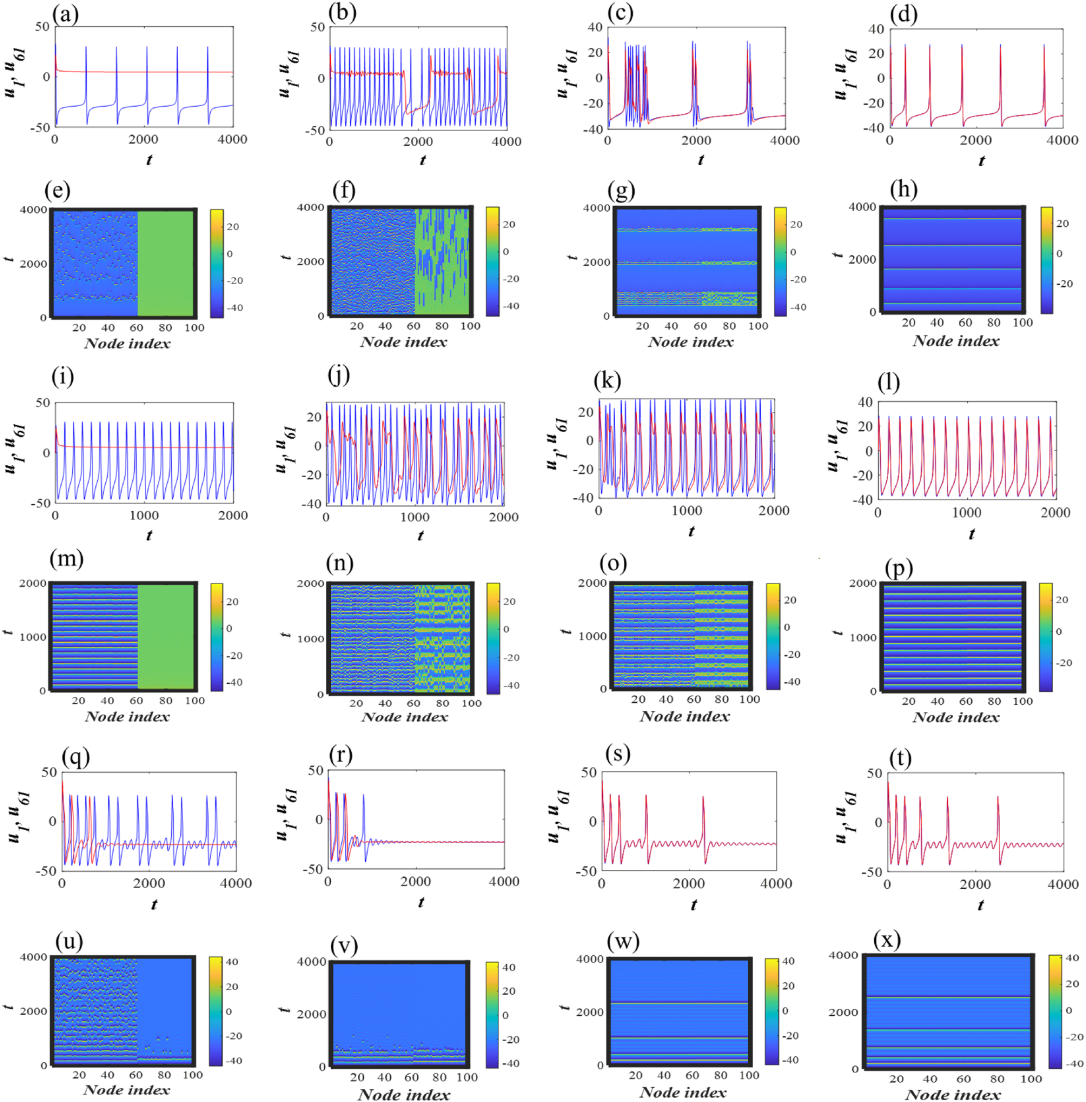
Time series and spatiotemporal dynamics of randomly connected network of class I & class II 2D M-L neurons (11) two different types of fractional exponents. First panel: (**a**–**d**) Set I: $\alpha _1 =\ldots= \alpha _{60}=1$ and $\beta _{61} =\ldots= \beta _{100}=0.75$ with $g_e=0.0001, 0.01, 0.08,1$. Third panel: (**i**–**l**) Set II: $\alpha _1 =\ldots= \alpha _{60}=1$ and $\beta _{61} =\ldots= \beta _{100}=0.75$ with $g_e=0.0001, 0.05, 0.08,1$. Fifth panel: (**q**–**t**) Set III: $\alpha _1 =\ldots= \alpha _{60}=0.86$ and $\beta _{61} =\ldots= \beta _{100}=0.81$ with $g_e=0.0001, 0.01, 0.5,1$. Corresponding spatiotemporal patterns are shown in second, fourth and sixth panels respectively. We have randomly picked two nodes from two sub-populations to plot the time signals. The time evaluation of one node marked with red line is chosen from the subpopulation having quiescent states (when $g_e=0$). The blue signal is chosen from the nodes which was kept at spiking states in the absence of coupling.

*m* number of nodes have identical fractional-order $\alpha$, reflecting oscillatory behavior and the remaining *n* nodes have fractional-order, $\beta$, reflecting excitable behavior. Thus, the total population size *N* can be expressed as $N=m+n$. First, we study the behavior of randomly connected class I excitable M-L neurons with two fractional-order exponents i.e., $\alpha =1$ and $\beta =0.75$. The total number of nodes in the network is $N=100$, and $m=60$ & $n=40$ i.e., we consider the network of *N* neurons with $60\%$ oscillatory and $40\%$ excitable neurons. In the absence of coupling ($g_e=0$), each oscillatory neuron in the network shows tonic spiking and the remaining neurons stay in quiescent state. With small increase in the electrical coupling $g_e=0.0001$, oscillatory subpopulation still remains in tonic spiking mode and another subpopulation shows quiescent state. The time signals of two randomly connected nodes from two subpopulations are marked with red and blue lines (see Fig. 5a). The red signal is randomly chosen from the quiescent nodes and blue signal from the spiking nodes. The spatiotemporal plot reveals that the spiking nodes (1–60) are desynchronized to each other (Fig. 5e). The system behavior changes if we increase the coupling 100 folds ($g_e=0.01$). Now, the subpopulation which was in quiescent state starts to exhibit bursting dynamics. Notably, the time interval of each burst is not periodic. Another subpopulation shows desynchronized irregular tonic spiking (see Fig. 5b, f). It is clear, if the coupling is increased in the mixed population, the periodic as well as quiescent nature vanishes and irregular bursting or spiking appears in the network. The entire network shows bursting dynamics with finite number of spikes in each burst with small increase of $g_e=0.08$ (see Fig. 5c, g). Here, two clusters with different amplitudes but the same phases are generated. Finally, at $g_e=1$, the coupled network exhibits almost synchronized behavior by changing the firing activity to tonic spiking (Fig. 5d, h).

Next, we increase the current stimulus $I=45$ and the oscillatory subpopulation shows fast spiking and the other subpopulation remains in quiescent state. At weak coupling ($g_e=0.0001$), the behavior of both subpopulations do not change. With the increase of coupling $g_e=0.05$ and 0.08, both the subpopulations start firing and show bursting dynamics. Finally, the activity of the random network changes to synchronized periodic spiking at $g_e=1$ (Fig. 5i–p). Next, we study the class II excitable M-L neurons in the same network architecture, i.e., one subpopulation is in quiescent state ($\beta =0.81$) and another subpopulation shows MMOs ($\alpha =0.86$) in the absence of coupling. At weak coupling, the neurons are in desynchronized state ($g_e=0.0001, 0.01$). Further increase of coupling, all the nodes in the network show spike frequency adaptation ($g_e=0.5$ and $g_e=1$). It is clear that each of the two sets of oscillators exhibits almost complete synchronization showing similar type of bursting with identical phases and amplitudes (see Fig. 5q–x).

### The reduced-order model with two subpopulations

**Figure 6:**
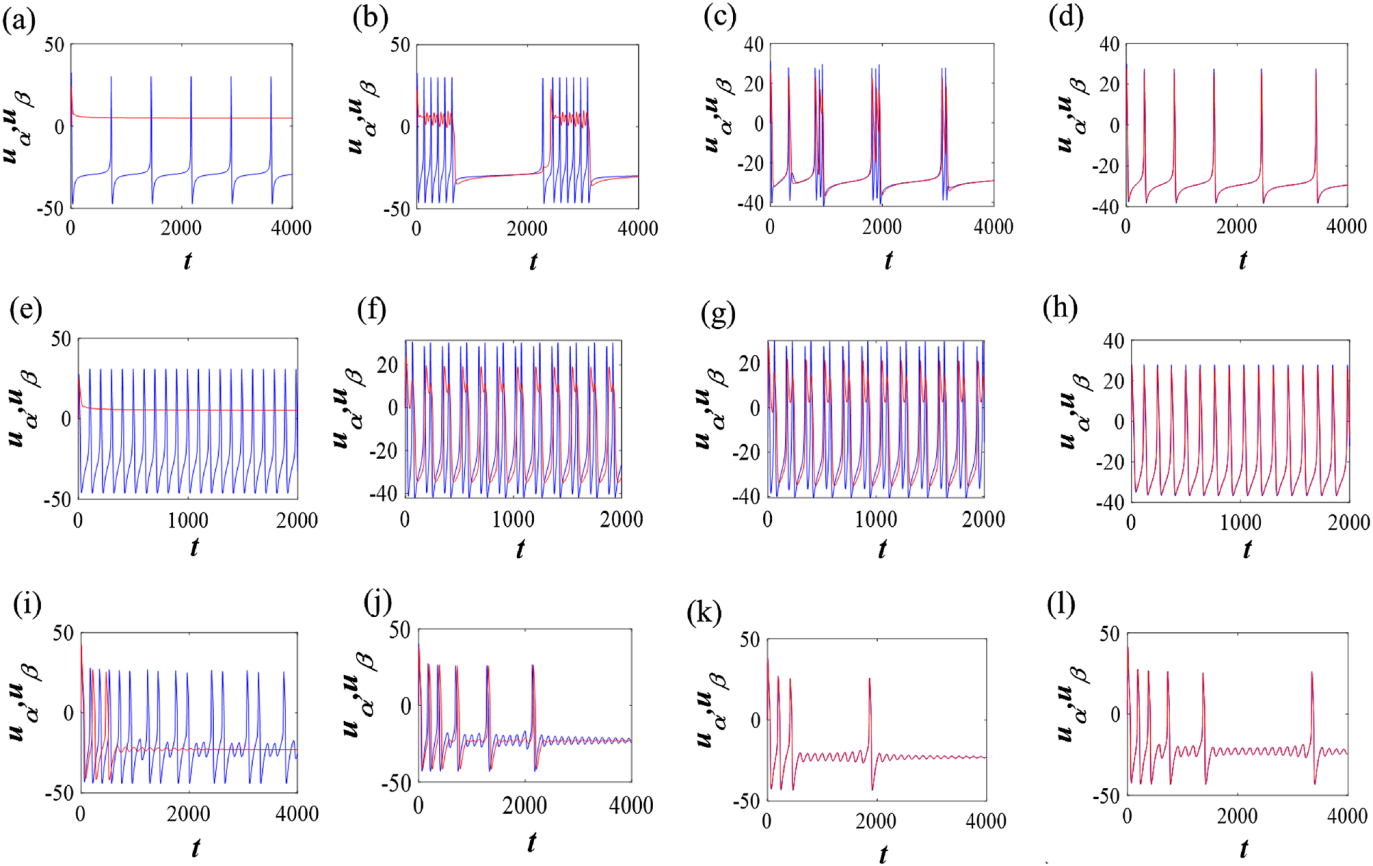
Evolution of neuronal responses for different coupling strengths of reduced-order two-coupled 2D M-L model (Eq. 12) for class I (set I & II) and class II (set III) excitable neurons with two different fractional exponents. (**a**–**d**) Set I: $\alpha =1,\, \beta =0.75$ and $g_e=0.0001,\, 0.01, \, 0.08,\, 1$ respectively. (**e**–**h**) Set II: $\alpha =1,\, \beta =0.75$ and $g_e=0.0001,\, 0.05, \, 0.08,\, 1$ respectively. (**i**–**l**) Set III: $\alpha =0.86,\, \beta =0.81$ and $g_e=0.0001,\, 0.01, \, 0.5,\, 1$ respectively.

In this section, a general and low-dimensional model description have been described to show that the reduced network exhibits the same feature as observed in the complete random network. In the intermediate coupling, we have observed a two cluster synchronization state that appears in the system. Motivated by this fact, we can write $u_1=u_2=u_3=\cdots =u_{60}=u_{\alpha }$, and $u_{61}=u_{62}=\cdots =u_{100}=u_{\beta }$. As we have considered the Erdös-Rényi graph, we can approximate the degree of each node/neuron by the average degree of the considered network^56–62^. Therefore, we can assume the degree of the *j* node $k_j=\langle k \rangle$. The number of spiking oscillators in the neighborhood of each oscillator is expected to be $(1-p_e)k=p_o k$ and the value will be $p_e k$ for the quiescent oscillators. Therefore, we can reconstruct a reduced-order model with two oscillators as follows

12 $$\begin{aligned} \begin{array}{l} C\frac{d^{\alpha }u_{\alpha }}{dt^{\alpha }} =- 0.5g_{Ca}(u_{\alpha }-V_{Ca})((1+\tanh (u_{\alpha }-V_1))/V_2)-v_{\alpha }{g_K}(u_{\alpha }-V_K)-g_L(u_{\alpha }-V_L)+I+g_e p_e(u_{\beta } - u_{\alpha }),\\ \frac{d ^{\alpha }v_{\alpha }}{d t^{\alpha }} =\phi \cosh ((u_{\alpha }-V_3)/2V_4)(0.5(1+\tanh ((u_{\alpha }-V_3)/V_4))-v_{\alpha }),\\ C\frac{d^{\beta }u_{\beta }}{dt^{\beta }} =- 0.5g_{Ca}(u_{\beta }-V_{Ca})((1+\tanh (u_{\beta }-V_1))/V_2)-v_{\beta }{g_K}(u_{\beta }-V_K)-g_L(u_{\beta }-V_L)+I+g_e p_o(u_{\alpha } - u_{\beta }),\\ \frac{d ^{\beta }v_{\beta }}{d t^{\beta }} =\phi \cosh ((u_{\beta }-V_3)/2V_4)(0.5(1+\tanh ((u_{\beta }-V_3)/V_4))-v_{\beta }), \end{array} \end{aligned}$$

where $p_e=\frac{n}{N}$ and $p_o=\frac{m}{N}$ represent the probabilities of excitable and oscillatory neurons respectively. We perform numerical simulations with the reduced order two coupled models, in which each subpopulation is captured by the identical fractional-order exponent exhibiting synchronous behavior for class I and class II M-L models. The numerical results suggest that the dynamics of the reduced-order model follows the same pattern as the entire graph when the two subpopulations follow cluster synchronization (see Fig. 6). For instance, bursting with two spikes can emerge for intermediate coupling in class I excitable system (Fig. 5k) separated by two clusters in the full network. Similar firing pattern exists in the reduced order model (Fig. 6g).

## Conclusions

In this study, first we focused on the single neuron’s dynamics that can change their response to various input statistics depending on the fractional exponents. We discussed the excitabilities of class I and class II conductance-based 2D M-L neurons that can be captured in realistic cortical neurons experimentally^2^. We examined corresponding bifurcation analysis of the classical-order model. The fractional exponent can trigger the firing variations that cannot be observed in the classical-order dynamics. The ability of the 2D M-L model for diverse spike responses including MMOs and MMBOs with fractional derivative is explored with spike frequency adaptation while decreasing the fractional exponent. The results demonstrate that the slow-fast 3D M-L in fractional domain provides various bursting patterns. It also changes its behavior from bursting to single train of spikes and fast spiking to irregular bursting for different suitable set of parameters that can not be captured in the classical-order model for a fixed set of parameters. The FOD shows an alternative representation of the spiking-bursting responses with the changes of fractional exponents in the dynamical models. The approach summarized the multiple behavior of the single excitable model to certain stimulus variance with a memory dependent activities. We investigated the role of electrical coupling in a random network, where certain fraction of nodes are in quiescent states. If the oscillatory nodes are in MMBOs, the entire population would exhibit spike frequency adaptation. On the other hand, if the uncoupled oscillatory nodes are kept at fast tonic spiking zone, the entire population split into two clusters revealing periodic bursting in intermediate coupling and at higher coupling all the nodes show tonic spiking. Motivated by the cluster synchronization phenomena, we were also able to reduce the network into two coupled dynamics, which successfully captured the dynamics of the entire network during cluster synchronization.

We established distinct effects on different membrane voltage features considering the fractional-order derivative. We showed that the differences in the neuronal characteristics are due to the memory effects. Fractional-order derivative provides rich dynamics and it is possible to explore realistic phenomena. These results demonstrate that the model and network provide a tractable approach to examine neuronal dynamics.

## Data Availability

All numerically simulated data generated or analysed during this study are included in this submitted article.

## Appendix: Numerical methods

In what follows, we present the numerical scheme used in our numerical simulations to approximate the solutions of the system of fractional-order differential equations of a variable $X \equiv (u,\,v)^\mathrm{T}$ described by

13 $$\begin{aligned} {\mathrm{{D}}^\alpha }\mathrm{X} = {f}\left( {\mathrm{X},\,t} \right) , \end{aligned}$$

where $\alpha \in ( 0,\, 1)$, $f \equiv (h_1,\,h_2)^\mathrm{T}$ and the Caputo-type derivative given by

14 $$\begin{aligned} {\mathrm{{D}}^\alpha }\left[ \begin{array}{l} u\left( t \right) \\ v\left( t \right) \\ \end{array} \right] = \frac{1}{{\Gamma \left( {1 - \alpha } \right) }}\int \limits _0^t {{{\left( {t - \tau } \right) }^{ - \alpha }}} \left[ \begin{array}{l} u'\left( \tau \right) \\ v'\left( \tau \right) \\ \end{array} \right] d\tau . \end{aligned}$$

The Caputo-type fractional-order derivative is consistent with the physical initial and boundary conditions. In this case, the firing characteristics of the system are strongly independent of the initial conditions^4,12^. We can define, the initial conditions for the fractional-order system that can be handled using an analogy with the classical-order derivative. It includes a memory effect with a convolution between the classical-order derivative and a power of time. It is efficient to integrate all the previous activities of the function weighted by a function that follows power law dynamics. The numerical simulations of the systems are performed using the time step $\Delta t=0.1$. The simulation results with a smaller time step do not show any significant differences. The bifurcation diagrams of the fixed points of the dynamical model were computed using the MatCont software package^63^. We simulated the model (Eq. 3) using the L1 scheme^4,12,18^ and approximated the fractional-order derivative as follows:

15 $$\begin{aligned} {\mathrm{{D}}^\alpha }\left[ \begin{array}{l} u\left( t \right) \\ v\left( t \right) \\ \end{array} \right] = \frac{{{{\left( {dt} \right) }^{ - \alpha }}}}{{\Gamma \left( {2 - \alpha } \right) }}\left[ \begin{array}{l} \sum \limits _{\mathrm{{k = 0}}}^{N - 1} {\left[ {u\left( {{t_{\mathrm{{k + 1}}}}} \right) - u\left( {{t_\mathrm{{k}}}} \right) } \right] \left[ {{{\left( {N - \mathrm{{k}}} \right) }^{1 - \alpha }} - {{\left( {N - 1 - \mathrm{{k}}} \right) }^{1 - \alpha }}} \right] } \\ \sum \limits _{\mathrm{{k = 0}}}^{N - 1} {\left[ {v\left( {{t_{\mathrm{{k + 1}}}}} \right) - v\left( {{t_\mathrm{{k}}}} \right) } \right] \left[ {{{\left( {N - \mathrm{{k}}} \right) }^{1 - \alpha }} - {{\left( {N - 1 - \mathrm{{k}}} \right) }^{1 - \alpha }}} \right] } \\ \end{array} \right] . \end{aligned}$$

The numerical solution of (13) can be formulated as (combining (13) and (15) )

16 $$\begin{aligned} \begin{array}{l} u\left( {{{ t}_N}} \right) \approx {\left( { dt} \right) ^\alpha }\Gamma \left( {2 - \alpha } \right) { h_\mathrm{{1}}}\left( {u(t),\,v(t)} \right) + u\left( {{{ t}_{N - 1}}} \right) \\ \,\,\,\,\,\,\,\,\,\,\,\,\,\,\,\, - \left[ {\sum \limits _\mathrm{k = 0}^{N - 2} {\left[ {u\left( {{{ t}_{k + 1}}} \right) - u\left( {{{ t}_k}} \right) } \right] \left[ {{{\left( {N - \mathrm k} \right) }^{\left( {1 - \alpha } \right) }} - {{\left( {N - 1 - \mathrm k} \right) }^{\left( {1 - \alpha } \right) }}} \right] } } \right] , \\ \end{array} \end{aligned}$$

17 $$\begin{aligned} \begin{array}{l} v\left( {{{ t}_N}} \right) \approx {\left( { dt} \right) ^\alpha }\Gamma \left( {2 - \alpha } \right) { h_\mathrm{{2}}}\left( {u(t),\,v(t)} \right) + v\left( {{{ t}_{N - 1}}} \right) \\ \,\,\,\,\,\,\,\,\,\,\,\,\,\,\,\, - \left[ {\sum \limits _\mathrm{k = 0}^{N - 2} {\left[ {v\left( {{{ t}_{k + 1}}} \right) - v\left( {{{ t}_k}} \right) } \right] \left[ {{{\left( {N - \mathrm k} \right) }^{\left( {1 - \alpha } \right) }} - {{\left( {N - 1 - \mathrm k} \right) }^{\left( {1 - \alpha } \right) }}} \right] } } \right] , \\ \end{array} \end{aligned}$$

where, ${t_\mathrm{k}}$ represents the $\mathrm {k}{\rm th}$ time step and ${t_\mathrm{k}} = \mathrm k\Delta t$. Hence, the numerical solution of (2) can be summarized as the difference between the Markov term weighted by the gamma function and the memory trace. The memory trace integrates all the past activities and it has no effect for $\alpha =1$. The nonlinearity in the memory trace increases as we decrease the fractional-order $\alpha$ from 1 and the system dynamics depends on time.
